# Developmental Changes in Neural Lateralization for Visual‐Spatial Function? Evidence From a Line‐Bisection Task

**DOI:** 10.1111/desc.70060

**Published:** 2025-08-09

**Authors:** Katrina Ferrara, Anna Seydell‐Greenwald, Catherine E. Chambers, Elissa L. Newport, Barbara Landau

**Affiliations:** ^1^ Center for Brain Plasticity and Recovery Georgetown University Washington DC USA; ^2^ Intellectual and Developmental Disabilities Research Center, Children's National Health System Washington DC USA; ^3^ Department of Cognitive Science Johns Hopkins University Baltimore Maryland USA

## Abstract

Studies of hemispheric specialization have traditionally cast the left hemisphere as specialized for language and the right hemisphere for spatial function. Much of the supporting evidence for this separation of function comes from studies of healthy adults and those who have sustained lesions to the right or left hemisphere. However, we know little about the developmental origins of lateralization. Recent evidence suggests that the young brain represents language bilaterally, with 4–6‐year‐olds activating the left‐hemisphere regions known to support language in adults as well as homotopic regions in the right hemisphere. This bilateral pattern changes over development, converging on left‐hemispheric activation in late childhood. In the present study, we ask whether this same developmental trajectory is observed in a spatial task, that is, strongly right‐lateralized in adults—the line bisection (or “Landmark”) task. We examined fMRI activation among children ages 5–12 years as they were asked to judge which end of a bisected vertical line was longer. We found that young children showed right‐lateralized activation in the same parietal and posterior temporal areas as has been shown among adults, with no significant effects of age on lateralization within the age range we tested. We discuss potential underlying mechanisms and suggest that understanding the development of lateralization for a range of cognitive functions can play a crucial role in understanding general principles of how and why the brain comes to lateralize certain functions.

**Summary:**

‐ Functional MRI was used to examine neural activation associated with a line bisection task in children ages 5−12 years.
‐ Children showed right‐lateralized activation in the same areas previously identified among adults.
‐ There were no effects of children's age on the degree of right‐lateralization.
‐ This illustrates stable lateralization over development for the line bisection task and contrasts with findings of increasing lateralization over age in the domain of language.

## 1 Introduction

Classically, it has been assumed that the two hemispheres of the brain are specialized for different functions: the left hemisphere for language and the right hemisphere for spatial functions (Bogen and Gazzaniga 1965; Broca 1861; Hugdahl and Westerhausen 2010; Lenneberg 1967; Sperry et al. 1969; Teuber 1974; Wernicke 1874). Abundant research has confirmed that language is, indeed, strongly left‐lateralized among healthy adults. A range of research has also supported the idea that spatial functions are right‐lateralized in adulthood. For example, adult patients with right‐hemisphere lesions (most often in the parietal lobe) often show striking impairments in the visual‐spatial domain, including hemispatial neglect (Bisiach and Luzzatti 1978; Vallar and Perani 1986; Vallar 1998) and difficulties in carrying out visual‐spatial construction tasks (Hecaen et al. 1956). Additional evidence suggests that individuals who have had perinatal strokes to the right hemisphere show poorer outcomes in spatial functioning than those who have had left hemisphere strokes (Stiles et al. 2012). Furthermore, individuals with left hemisphere perinatal strokes may show deficits in spatial tasks when language is re‐organized to the right hemisphere, possibly compromising right‐localized spatial functions (Lidzba et al. 2006).

The idea that the brain's hemispheres exhibit specialization of function is rooted in the general hypothesis that it is most efficient for each hemisphere to specialize in different kinds of processing (Cai et al. 2013; Gerrits et al. 2020; Levy 1977; Vallortigara 2006). In the domain of language, some have proposed that small biases in processing preferences may lead to larger system‐wide preferences, with the right hemisphere biased toward processing characteristics of pitch (Zatorre and Belin 2001; Zatorre et al. 1992) or, more generally, aspects of the speech signal that span larger temporal windows (Poeppel 2003). These initial biases could then lead to the division of labor between phonetics (typically left‐lateralized) and prosody (typically right‐lateralized; Zatorre and Gandour 2008; Seydell‐Greenwald et al. 2020). In the domain of space, analogous proposals have been offered. For example, Ivry and Robertson (1998) proposed that the right hemisphere is biased to process low‐frequency visual information, leading to “global” spatial processing preferences, while the left hemisphere is biased to process high‐frequency information, leading to “local” processing preferences (see also Kosslyn et al. 1989).

In particular, there is one experimental paradigm that provides an abundance of evidence supporting the right hemisphere's specialization for spatial ability: This is the classic task known as the line bisection task and its perceptual version, the “Landmark Task” (Fink et al. 2000). Traditionally used for assessing hemispatial neglect in clinical settings (e.g., Jansen et al. 2005; Karnath 1988; Vallar and Perani 1986), this task has also been widely used to study lateralization of visual‐spatial function in healthy adults. In the classic line bisection task, participants are asked to draw a hashmark at the perceived center of a horizontal line. In the corresponding Landmark Task, participants are asked to judge whether a short vertical line correctly bisects a horizontal line or whether it deviates from the midpoint. People with disrupted right parietal function (either due to lesion or transcranial magnetic stimulation) deviate to the right side of the line, presumably because they neglect the contralesional left side of space, representing that side as smaller than it really is (Fierro et al. 2000; Karnath 1988; Schenkenberg et al. 1980). In healthy adults, neuroimaging studies using versions of the Landmark and line bisection tasks have revealed strong activation in right parietal cortex (Cavézian et al. 2012; Çiçek et al. 2009; Fink et al. 2000; Foxe et al. 2003; Waberski et al. 2008). Indeed, the task has high reliability, showing reproducible right hemispheric dominance in 93% of adult participants (Schuster et al. 2017). The strong tendency for this task to show right lateralization may be the reason why researchers have used it as a proxy of sorts for visual‐spatial functions as a whole, particularly in studies that examine whether lateralization of language and space are organized across the hemispheres in complementary fashion (Badzakova‐Trajkov et al. 2010; Badzakova‐Trajkov et al. 2016; Cai et al. 2013; Flöel et al. 2005; Jansen et al. 2005; Rosch et al. 2012).

Interestingly, lateralization of activation to the right hemisphere that is associated with the line bisection task is not limited to horizontal presentation of the line. Fink et al. (2001) directly compared fMRI activation for line bisection performed on horizontal versus vertical lines within the same healthy adult participants. They observed right‐lateralized activation in posterior parietal cortex regardless of stimulus orientation, concluding that “orientation did not differentially affect the neural mechanisms underlying the visuospatial judgment per se” (Fink et al. 2001, S65–S66). Furthermore, no significant interactions between task (bisection judgment vs. control) and stimulus orientation (horizontal vs. vertical) were found. In a recent adaptation of the task that uses a vertical line with a horizontal bisector, strong right‐lateralized parietal activation was also found in healthy adults at both the group and individual level (Seydell‐Greenwald et al. 2019).

An important starting point for the present study is the recognition that, although there is robust evidence that the line bisection task is strongly right‐lateralized in healthy adults, this may not hold true for all spatial functions. Indeed, lateralization patterns vary significantly across different spatial tasks, even among healthy adults. Perhaps surprisingly, research over the past decade has shown that some basic spatial‐cognitive functions appear to be bilateral in healthy adults. For example, one classic spatial function—mental rotation—activates critical regions of both hemispheres to a similar degree (Carpenter et al. 1999; Cohen et al. 1996; Desrocher et al. 1995; Jordan et al. 2002; Kosslyn et al. 1998; Peronnet and Farah 1989; Richter et al. 1997; Tagaris et al. 1996). Two recent meta‐analyses of studies using mental rotation tasks support this (Tomasino and Gremese 2016; Zacks 2008). Similar findings have been shown for other widely‐used spatial tasks. For example, visual‐spatial construction, as tested by a puzzle task inspired by the well‐known WASI‐II Block Design task (Wechsler, 2011) shows consistent activation of bilateral inferior and superior parietal regions in healthy adults (Seydell‐Greenwald et al. 2017) and children (Ferrara et al. 2020; see Ebner et al. 2011, for similar results).

In contrast to the rich history and growing number of neuroimaging studies on hemispheric lateralization in adults, we know remarkably little about the developmental origins of hemispheric specialization. Importantly, research on the development of language is revealing a complex process in which adult lateralization of function is only the endpoint. By 4 months of age, infants show some degree of left lateralization of function for speech perception (Dehaene‐Lambertz et al. 2002; Petitto et al. 2012; Perani et al. 2011). But there is also evidence that lateralization for language changes considerably over childhood. In healthy children, the degree of left lateralization for sentence and word processing increases significantly between the ages of 4 and 13; younger children show much more bilateral activation for sentences, with increasing left lateralization over age; the adult pattern of strong left lateralization is not reached until adolescence (Berl et al. 2014; Olulade et al. 2020). These findings may help to explain the unusually robust preservation of language in individuals who have sustained perinatal strokes: if both the left and right hemispheres are initially capable of representing language, then damage to the left hemisphere should leave the healthy right hemisphere to support language (Newport et al. 2017; Olulade et al. 2020). This point was originally made by Lenneberg (1967), who emphasized the high degree of plasticity in the young brain. Subsequent studies have documented the resiliency of language after perinatal stroke (Newport et al. 2017; Stiles et al. 2012). In the spatial domain, we know less about the typical developmental course of lateralization, but the findings for language raise the possibility that a similar pattern might be found, with early bilaterality for some spatial functions followed by increased lateralization toward one hemisphere (presumably the right). The present study examines this possibility, focusing on a task that is well‐known for evoking right‐lateralized brain activation in adults: the line bisection judgment or “Landmark Task” (Fink et al. 2000).

Developmental evidence for patterns of lateralization associated with spatial tasks is quite limited, and the findings are mixed, with some studies finding evidence of right‐lateralized function and others finding bilateral activation. Lidzba et al. (2013) used a complex visual search task that involved side‐by‐side comparison of two Rey‐Osterrieth Complex Figures (Rey 1941). Participants were required to indicate whether one detail was missing from one of the figures. Interestingly, individuals who were high‐performers on the behavioral task in the scanner (regardless of age) had stronger activation in right superior parietal cortex, suggesting a more mature visual search network that is right‐lateralized. Everts et al. (2009) used the same task and also evaluated performance on the Rey‐Osterrieth Complex Figure outside of the scanner (participants were required to draw reproductions of the figure as accurately as they could). Performance on this task outside of the scanner was found to correlate with the degree of right‐lateralized activation during the in‐scanner visual search task. Using the same task with 8−20‐year‐olds, Everts et al. (2009) found right lateralization of parietal and frontal activation that increased with age. These studies suggest that the right hemisphere plays a privileged role in some aspects of spatial processing, which may become further strengthened as children develop. Conversely, the opposite developmental pattern is found in some studies that used different spatial tasks. Kucian et al. (2007) found right‐lateralized activation in children during a mental rotation task, and bilateral activation in adults. Nagel and colleagues measured brain activation in 10−16‐year‐olds during a spatial working memory task and found that older participants showed greater bilateral activation in posterior parietal cortex than younger participants (Nagel et al. 2013). Still other studies have found that children show consistent bilateral processing of spatial information. For example, Ebner et al. (2011) asked participants to determine whether an abstract, colored shape could fit with another “like a piece in a puzzle.” Activation in right and left superior parietal areas was reported in participants between 7 and 17 years of age (no age effects were evaluated). Lastly, Ferrara et al. (2020) tested children between the ages of 5 and 11 and found consistent and robust bilateral parietal activation associated with a visual‐spatial construction task.

In sum, the little we know about the developmental course of lateralization for spatial functions suggests that the pattern is mixed, with some tasks consistently activating bilateral regions of the parietal lobe from early childhood and into adolescence and adulthood, and others appearing to show patterns of right lateralization that are linked with maturation and performance. These mixed results are likely in part due to the different computational requirements of the different tasks. This highlights how little is known about the origins and developmental trajectories for neural lateralization of spatial tasks, which may vary depending on the computational requirements of the functions involved.

Here we aim to discover whether certain spatial functions may show a developmental lateralization pattern that is similar to what has been found for language (i.e., initially bilateral and becoming increasingly lateralized to the hemisphere that is dominant for the task in adulthood). This calls for a task that is definitively right‐lateralized among healthy adults. Fortunately, a number of neuroimaging studies provide a strong basis for the conclusion that the line bisection task is consistently right‐lateralized in adults. This suggests that it is a good candidate for testing whether the developmental trajectory for lateralization of spatial function can, in principle, parallel the trajectory found in language.

Our understanding of the neural underpinnings of the line bisection and/or Landmark Task in early development is, at present, quite limited. The classic line bisection task has been used to study visual‐spatial attention, spatial acuity, and neglect in both typically and atypically developing children (e.g., Bradshaw et al. 1987, Bradshaw et al. 1988; Chokron and De Agostini 1995; Dellatolas et al. 1996; Failla et al. 2003; Ferro et al. 1984; Hausmann et al. 2003; Johnston and Shapiro 1986; Paquier et al. 1999; Patro et al. 2018; Saj et al. 2020; Sheppard et al. 1999, Sheppard et al. 2002). Although these behavioral studies alone cannot address the issue of lateralization or its developmental profile, they can provide a picture of developmental change in accuracy. In perhaps the most comprehensive investigation of behavioral performance, van Vugt et al. (2000) tested 650 typically developing children aged 7−12 years on both horizontal and vertical versions of the task. Participants were instructed to mark the midpoint of the line with their preferred hand using a pen. Performance systematically improved with age, with older children more accurately marking the midpoint of the line than younger children. Recently, Hoyos et al. (2020) used a perceptual version of the horizontal line bisection task to understand the development of frontal‐parietal attentional networks. In this behavioral task, they found that participants identified the bisection point in an overall leftward direction. This leftward bias was found among all ages (6−14 years), largest in the youngest children (grades 1−3) and decreasing among older children (grades 6−8), who showed the same very small bias as adults (i.e., close to 0).

In the present study, we used functional magnetic resonance imaging (fMRI) to examine the lateralization trajectory for a version of the line bisection/Landmark Task. We adapted an experimental paradigm from previous work that is suitable for use in the scanner and has shown robust right‐lateralized parietal activation in healthy adults (Seydell‐Greenwald et al. 2019). We asked whether this task also shows the same pattern in children between ages 5 and 12, or whether, in parallel with findings in the language domain, the youngest children show a predominantly bilateral pattern of activation that becomes right‐lateralized in the oldest children. If the developmental profile for lateralization shows a pattern similar to that found for language, these findings would suggest a common mechanism of hemispheric specialization that holds for different domains of cognitive function.

## 2 Materials and Methods

### 2.1 Participants

The data presented here are from 36 typically developing children between the ages of 5 and 12 (5.88−12.64 years, Mean age = 8.92 years, SE = 0.32 years, 21 females). An additional five children participated in the study, but their data were excluded due to excessive motion in the scanner (greater than 3 mm in any direction). Participants were recruited via local parent groups and preschools and were compensated for their time. All participants had normal or corrected‐to‐normal vision with no history of neurological impairment or abnormality, and all were fluent English speakers. The study was approved by the university Institutional Review Board. Informed consent was provided by parents, and written assent was provided by all children before the study procedures began.

### 2.2 Stimuli, Design, and Procedure

**FIGURE 1:**
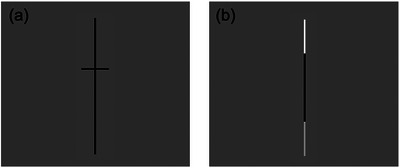
Experimental design. (a) Spatial condition: participants indicated whether the bottom or top part of the line was longer. (b) Luminance condition: participants indicated whether the bottom or top part of the line was brighter.

Participants performed a visual‐spatial line bisection task while lying on their backs in the scanner. They were presented with a vertical line at the center of the screen that was divided into two segments and were asked to report which of the two segments was longer (Figure 1). The task is similar to that used by Hoyos et al. (2020), eliminating the traditional manual measure of line bisection and instead presenting participants with a line that is bisected slightly off‐center and asking them to make judgements of relative length of the two line sections. We opted to use vertical lines instead of horizontal for several reasons. First, as reviewed above, it has been shown that this task elicits robust right‐lateralized parietal activation, regardless of whether the stimulus lines are presented in vertical or horizontal orientations (Fink et al. 2001). Second, we aimed to build upon the findings of a previous study of adults (Seydell‐Greenwald et al. 2019), which also used vertical lines. Third, as reviewed by Seydell‐Greenwald et al. (2019), using a vertical line presented at screen center minimizes activations associated with shifts in spatial attention to left or right hemispace, which could influence lateralization of activation patterns (Corbetta et al. 1993; Corbetta and Shulman 2011; Heilman and van den Abell 1980; Mesulam 1981). Thus, we are able to isolate right‐lateralized activation associated with the spatial computations involved in comparison of relative length and proportion, without also drawing in activation that may be associated with left/right shifts in attention.

The present study included a Spatial condition and a Luminance condition (Figure 1). In the Spatial condition, a vertical line was presented with a small horizontal line segmenting it roughly in half. Participants were asked to decide which portion of the line was longer, either the top portion above the bisector or the bottom portion below the bisector. In the Luminance condition, which served as the control condition, the same vertical line was presented, but now the ends were different shades of grey. Participants were asked to decide if the top or the bottom part of the line was brighter. Both conditions thus required comparison of the ends of the vertical line, ensuring that any shifts of spatial attention would contribute to activation to a similar degree. The Luminance control condition was intentionally designed to be highly visually similar to the Spatial condition, but importantly, it did not involve spatial judgements of relative length. Comparison of the Spatial and Luminance conditions should therefore reveal the critical cortical areas that are uniquely involved in the Spatial condition. Both the Spatial and Luminance conditions were calibrated to produce high accuracy. This was done to avoid the potential influence that task difficulty can have on lateralization of neural activation (Just et al. 1996; Murphy and Garavan 2004; Yeatman et al. 2010). By choosing stimuli for which judgments would be relatively easy, we achieved high and similar levels of accuracy for the two conditions while keeping our participants actively engaged across a wide age range (see Ferrara et al. 2020 for a similar method).

Following consenting and MRI safety screening, participants were shown the task, called “The Line Game,” on a laptop outside of the scanner. They then completed a 20‐minute training session to familiarize them with the scanning environment, first in a fabric play tunnel and then in a mock scanner.

In the scanner, visual stimuli were projected onto a screen and viewed by participants through a slanted mirror mounted on the head coil. Stimuli were vertical black lines presented on a gray background (RGB 127, 127, 127). The lines were 8 cm long and 1 mm thick on the computer screen, corresponding to a length of 6.4° visual angle and a thickness of 0.08° visual angle given the projection magnification and effective viewing distance. For the Spatial condition, each vertical line was bisected by a short horizontal line (length: 128°, thickness: 0.08°). The bisector was located 0.8° (12.5% of the line length) above or below the veridical line center. For the Luminance condition, there was no bisecting line, but the uppermost and lowermost tips (length: 1.6°) of each vertical line were lighter than the rest of the line. One of the tips was white (RGB 255, 255, 255) and the other was gray (RGB 190, 190, 190). To prevent participants from basing their judgments on the location of the line tips on the screen, the vertical position of the stimuli on the screen was varied randomly within a range of 2.4° around screen center. To prevent participants from comparing lines across trials, a longer, dashed line (length: 12.64°) was presented during the 200‐ms inter‐stimulus interval to visually mask the preceding stimulus and any potential afterimages. Mask and location randomization were applied to all conditions to keep visual stimulation as similar as possible across tasks.

Both the Spatial and Luminance conditions required participants to push one of two buttons. Button number 1 was held in the left hand, and button number 2 was held in the right hand. Participants pushed button number 1 when they judged that the line was longer on the top (in the Spatial condition) and when they judged that the line was brighter on the top (in the Luminance condition). Participants pushed button number 2 when they judged that the line was longer or brighter on the bottom in the respective conditions.

### 2.3 Functional MRI Paradigm

Participants completed four 3‐min runs of “The Line Game.” Each run contained two blocks of the Spatial condition and two blocks of the Luminance condition; order of presentation was counterbalanced across the four runs. Blocks were 24 s long and each block was followed by a 9‐s fixation rest period. To remind participants of the different tasks for the two conditions, recorded auditory instructions were presented at the start of each block (9 s). At the start of Spatial blocks, participants heard a voice that asked, “Is the line longer on the top or the bottom?” At the start of the Luminance blocks, participants were asked “Is the line brighter on the top or the bottom?” These instructions were accompanied by an image of a cartoon giraffe or a polar bear with a thought bubble containing examples of the stimuli from the relevant condition (see Figure S1).

Two Cedrus fiber optic button boxes were used to record participant responses. All trials were self‐paced; participants completed as many trials as they could within each block. This ensured that children were not rushed while at the same time preventing idle periods between trials. The next trial appeared immediately following a button push or if a response had not occurred within 3 s. Piloting indicated that this response time (RT) limit was generous even for the youngest participants.

### 2.4 Image Acquisition

A research‐dedicated Siemens Trio Tim 3‐Tesla magnetic resonance imaging scanner with a 12‐channel birdcage head coil was used to acquire imaging data. Participants’ heads were stabilized with foam padding, and they wore headphones mounted in Bilsom ear defenders. This protected them from the scanner noise and enabled them to hear the auditory recordings played during the instruction periods. Visual stimuli were presented using E‐Prime 2.0 and were projected onto a screen at the back of the scanner via an Epson PowerLite 5000 projector, which participants were able to view via a mirror mounted on the head coil.

A low‐resolution anatomical image to aid volume placement for subsequent scans was first acquired during a 1‐min localizer scan. Four 3‐min functional runs were next acquired to measure blood‐oxygen‐level‐dependent (BOLD) signal changes associated with the Spatial and Luminance conditions. Functional images were acquired with a gradient echo‐planar T2* sequence (50 horizontal slices acquired in descending order, voxel size 3 × 3 × 2.8 mm^3^ with a distance factor of 7% between slices, TR = 3 s, TE = 30 ms, flip angle = 90 degrees, matrix 64 × 64, duration 3 min, 60 volume acquisitions). Lastly, a high‐resolution structural scan was acquired, during which children watched a movie. Structural T1‐weighted images were acquired using magnetization‐prepared rapid‐acquisition gradient echo (MPRAGE) (176 sagittal slices, voxel size 1 × 1 × 1 mm, TR = 2530 ms, TE = 3.5 ms, inversion time (TI) = 1100 ms, flip angle = 7 degrees, matrix 256 × 256, duration 4 min).

### 2.5 Imaging Analysis

#### 2.5.1 Preprocessing

Brain Voyager QX software (Brain Innovation, Maastricht, the Netherlands) was used to conduct the imaging analyses. Anatomical data underwent inhomogeneity correction and transformation into Talairach space using a nine‐parameter affine transformation. Manual definition of the landmarks was performed for the Talairach transformation. Functional data were preprocessed, including removal of the first two volume acquisitions to allow for T1 saturation, slice scan time correction, linear trend removal, 3D motion correction using rigid‐body transformation, co‐registration to the anatomical scan using 9‐parameter gradient‐based alignment, and spatial smoothing with a 6 mm full‐width at half‐maximum (FWHM) Gaussian kernel.^1^ Functional data were transformed into Talairach space using the same transformation applied to the anatomical data.

#### 2.5.2 Statistical Analysis

Voxel time courses from the four functional runs were combined and fitted with a general linear model (GLM) to investigate whole‐brain activation. Rest periods served as the model's baseline. The GLM contained two condition predictors: one for Spatial blocks and one for Luminance blocks. The time course for each predictor was determined by convolving the time course of the stimulation (a boxcar predictor that was “on” during the condition and “off” otherwise) with the hemodynamic response function (two gamma HRF, time to peak 5 s, time to undershoot peak 15 s). The model also contained a predictor for the instruction periods that occurred at the start of each block, z‐transformed motion estimates, and a constant predictor for each functional run as nuisance regressors. Voxel time courses were normalized (percentage signal change transformation) and corrected for serial autocorrelations (second‐order model). For group‐level analyses, beta maps for all participants were combined into a random effects (RFX) analysis. For single‐subject analyses, data from a given participant's four functional runs were combined in a fixed effects (FFX) analysis. All activation maps were thresholded using a single‐voxel threshold of *p* < 0.001 in combination with a cluster‐size threshold of *k* < 0.05 (as determined by Monte‐Carlo simulation with 1000 iterations, using the Cluster‐Level Statistical Threshold Estimator plugin for BrainVoyager QX).

#### 2.5.3 Regions of Interest (ROIs)

In addition to analyses of whole‐brain activation, we also focused on activation within specific ROIs. We defined ROIs using two different methods: the first followed an anatomical definition approach and the second followed a functional definition approach. First, we created an anatomically‐defined bilateral posterior parietal ROI that was made up of Brodmann Areas (BAs) 7, 40, and 39 (see Figure S2). These BAs are commonly cited in the literature as important for visual‐spatial processing. This anatomical ROI has been used in previous studies to analyze lateralization of visual‐spatial function in healthy adults (Seydell‐Greenwald et al. 2017, 2019) and healthy children (Ferrara et al. 2020). We also followed a second approach, using a functionally‐defined ROI derived from independent adult data (these results are reported in the Supplementary Information).

#### 2.5.4 Laterality Indices (LIs)

We investigated lateralization of brain activation within the two ROIs described above to determine whether activation occurs more on the right than the left. We employed a measure commonly used in the neuroimaging literature on language, the lateralization index (LI), which quantifies activation in the left and right hemispheres and then computes the ratio of left versus right (left activation—right activation)/(left activation + right activation). An LI of −1 indicates complete lateralization to the right hemisphere, and an LI of 1 indicates complete lateralization to the left hemisphere.

An in‐house script (Matlab 2015b and BVQXtools v0.8d) was used for LI computation and mask generation. A bootstrapping approach was used to compute a weighted mean of LIs obtained at different activation thresholds (Wilke and Lidzba 2007; Wilke and Schmithorst 2006). Bootstrapping is a statistical technique whereby multiple resamples are taken from an original sample “in order to estimate a bootstrap distribution that allows approximating the ‘real’ distribution of the original sample” (Davison and Hinkley 1997, 522; Hesterberg et al. 2005; Janssen and Pauls 2003; Moore et al. 2003; Wilke and Schmithorst 2006). Twenty‐five different *t*‐threshold values were applied to the activation maps, ranging in equal intervals from 0.1 to the maximum t‐value in the participant's map. To prevent distortion of the results by outliers, LI computation was aborted for thresholds at which there were fewer than 10 active voxels on either side. Otherwise, 10,000 LI estimates were computed from the sum of *t*‐values from randomly‐drawn active voxels on each side (0.25 of all active voxels were sampled per estimate). A trimmed mean (including only the central 50% of estimates) of the 10,000 estimates was used to provide a robust LI estimate that limits the influence of statistical outliers. Following LI computation at each threshold, each LI was multiplied by its *t*‐threshold value, such that LIs for stricter thresholds received higher weights. This approach enables us to account for the “meaningfulness” of voxels obtained at different thresholds, because voxels that are significant at higher thresholds have a higher correlation with the task (Wilke and Schmithorst 2006). Lastly, the sum of all weighted LIs was divided by the sum of all *t*‐thresholds.

## 3 Results

### 3.1 Behavioral Performance

**FIGURE 2:**
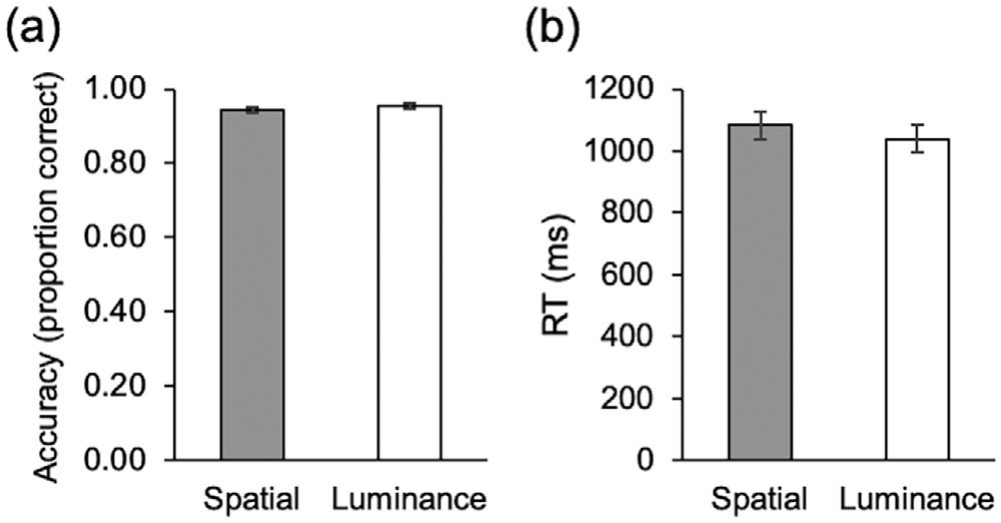
Behavioral performance on the “Line Game.” (a) Average proportion of correct responses for the Spatial and Luminance conditions. (b) Average reaction time (RT) for the Spatial and Luminance conditions. Error bars represent ± standard error of the mean.

Overall, children showed highly accurate task performance in both conditions (Figure 2). The Spatial and Luminance conditions did not significantly differ in terms of accuracy (proportion of correct responses) (Spatial: *M* = 0.94, SE = 0.01; Luminance: *M* = 0.95, SE = 0.01, *t*(35) = −0.65, *p* = 0.52, 95% confidence interval [CI] = [–0.02, 0.01]) or RT (Spatial: *M* = 1082.47, SE = 45.70; Luminance: *M* = 1039.26, SE = 47.38, *t*(35) = –0.03, *p* = 0.98, 95% CI = [−92.41, 89.92]). No gender differences for accuracy or RT were found (*p*’s > 0.41).

Pearson product‐moment correlation coefficients (two‐tailed) were computed to explore relationships among age, accuracy, and RT. Accuracy in each condition was not significantly correlated with age (Spatial: *r* = 0.19, *p* = 0.26; Luminance: *r* = 0.26, *p* = 0.17). The same was found for RT in each condition (Spatial: *r* = –0.06, *p* = 0.72; Luminance: *r* = –0.26, *p* = 0.23). Within each condition, RT and accuracy were not significantly correlated, indicating that participants who were quicker to respond were not necessarily more accurate (Spatial: *r* = –0.06, *p* = 0.74; Luminance: *r* = 0.06, *p* = 0.71). Overall, analyses of behavioral performance demonstrate that the task was appropriately designed and could be completed by children across the age range.

### 3.2 Imaging

We first carried out whole‐brain group analyses to determine the areas of significant activation for the Spatial condition compared to the Luminance condition. We then investigated developmental changes in lateralization patterns of activation.

#### 3.2.1 Areas of Activation: Whole Brain and ROI Analyses

**FIGURE 3:**
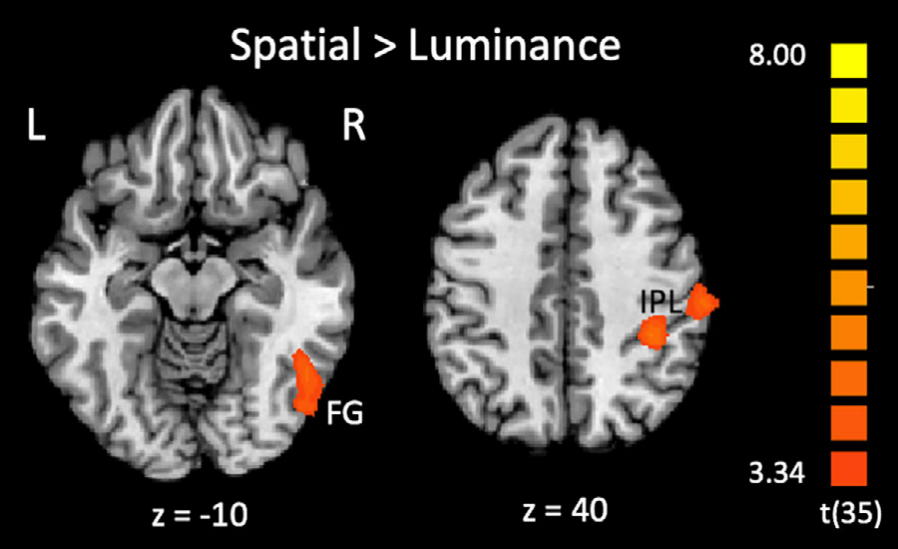
Group‐level activation map across all child participants: Areas displaying significantly stronger activation for the contrast of Spatial > Luminance for the line bisection task. Activation was observed in the right hemisphere in the fusiform gyrus (FG, BA 37) and in the inferior parietal lobule (IPL, BA 40) in two clusters. Activation maps are overlaid on the Colin27 brain template transformed into Talairach space and thresholded at *p* < 0.001 single‐voxel threshold combined with a *k* < 0.05 cluster‐size threshold. Activation details can be found in Table 1.

Whole‐brain GLM contrasts were conducted to identify areas that showed stronger activation for the Spatial condition compared to the Luminance condition (Spatial > Luminance) (Table 1 and Figure 3). In this group analysis, children showed an activation peak in the right hemisphere in the fusiform gyrus (FG) and two peaks in the right hemisphere in the inferior parietal lobule (IPL).^2^

**TABLE 1:** Activation clusters from the whole‐brain analysis of all participants, Spatial > Luminance.

Location description	BA	Peak Tal cords.	Center of gravity	Peak *t* value	Average *t* value	Average *p* value	Cluster extent (mm^3^)
Fusiform gyrus	37	52, −73, −5	48, −62, −6	4.97	3.82	0.000754	3538
Inferior parietal lobe	40	36, −37, 40	36, −36, 41	4.95	3.83	0.000742	1310
Inferior parietal lobe	40	60, −22, 37	57, −24, 39	4.52	3.75	0.000836	1081

We next sought to examine this response profile in greater depth. To investigate the response activation profile within an ROI, the average percentage signal change across all voxels in the ROI was extracted separately for each child participant and condition (thresholded at *p* < 0.001 single‐voxel threshold combined with a *k* < 0.05 cluster‐size threshold). This was done separately for the left and the right hemispheres to evaluate potential differences in activation. Multiple linear regressions were calculated to predict activation (percentage signal change) based on age, accuracy, and RT for behavioral performance in the scanner. Separate linear regressions were calculated for the left hemisphere and for the right, in light of the possibility that activation may be significantly related to age or performance in one hemisphere but not the other.

For the anatomically‐defined parietal ROI, none of the regression analyses were significant, not for the Spatial condition in the right hemisphere, *F*(3, 32) = 1.24, *p* = 0.312, with an *R* ^2^ of 0.10 (see Table 2 for significance test results for Age, Accuracy, and RT as predictors of activation), nor for the Spatial condition in the left hemisphere, *F*(3, 32) = 1.30, *p* = 0.293, *R* ^2^ of 0.11, nor for the Luminance condition in the right hemisphere, *F*(3, 32) = 0.43, *p* = 0.734, *R* ^2^ of 0.04, nor for the Luminance condition in the left hemisphere, *F*(3, 32) = 0.53, *p* = 0.668, *R* ^2^ of 0.05.

**TABLE 2:** Summary of the linear regression equation for variables predicting activation (percentage signal change) for the Spatial condition in the parietal anatomically‐defined ROI in the right hemisphere.

Variable	*B*	SE B	*β*	*p*
Age	0.047	0.038	0.264	0.218
Accuracy	<0.001	<0.001	0.394	0.069
RT	0.346	1.129	0.051	0.761

*Note*: Age was not a significant predictor of activation.

Neither age, nor accuracy or RT were significant predictors of activation for either condition in either hemisphere (for parallel analyses using the adult‐defined functional ROI, see Supplementary Information). Overall, these findings suggest that right‐lateralization of activation for the line bisection task is stable across the age range tested here.

#### 3.2.2 LIs

**FIGURE 4:**
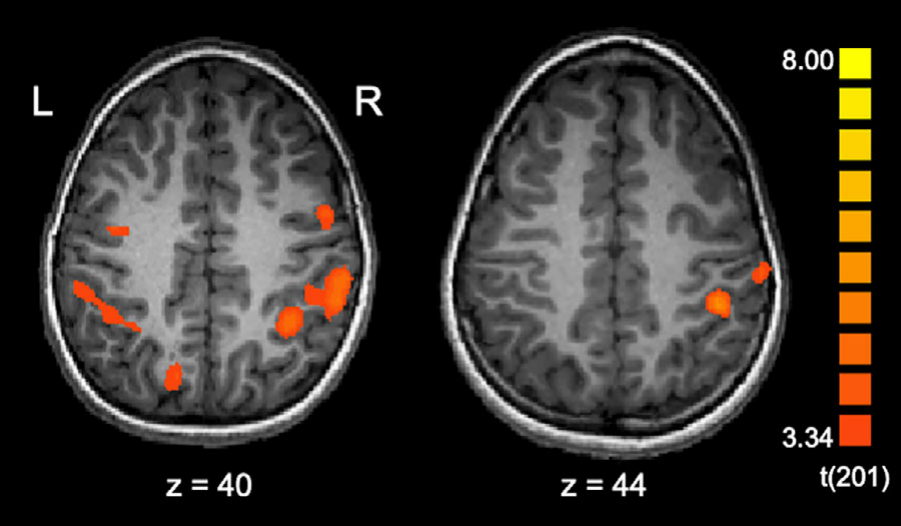
Individual participant activation patterns (contrast: Spatial > Luminance). Activation maps are overlaid on the individual's MPRAGE, transformed into Talairach space, and thresholded at *p* < 0.001 single‐voxel threshold combined with a *k* < 0.05 cluster‐size threshold. See Figure S4 for additional examples of individual activation maps.

We next investigated whether the profile of lateralization for the line bisection task changes with age. As a first step, we carried out whole‐brain analyses for individual child participants, examining children's activation for the Spatial > Luminance contrast. Inspection of the individual participant maps revealed great interindividual variability, with some participants showing bilateral and others showing right‐lateralized activation. Figure 4 shows examples of these individual activation patterns (see Figure S4 for additional examples).

**FIGURE 5:**
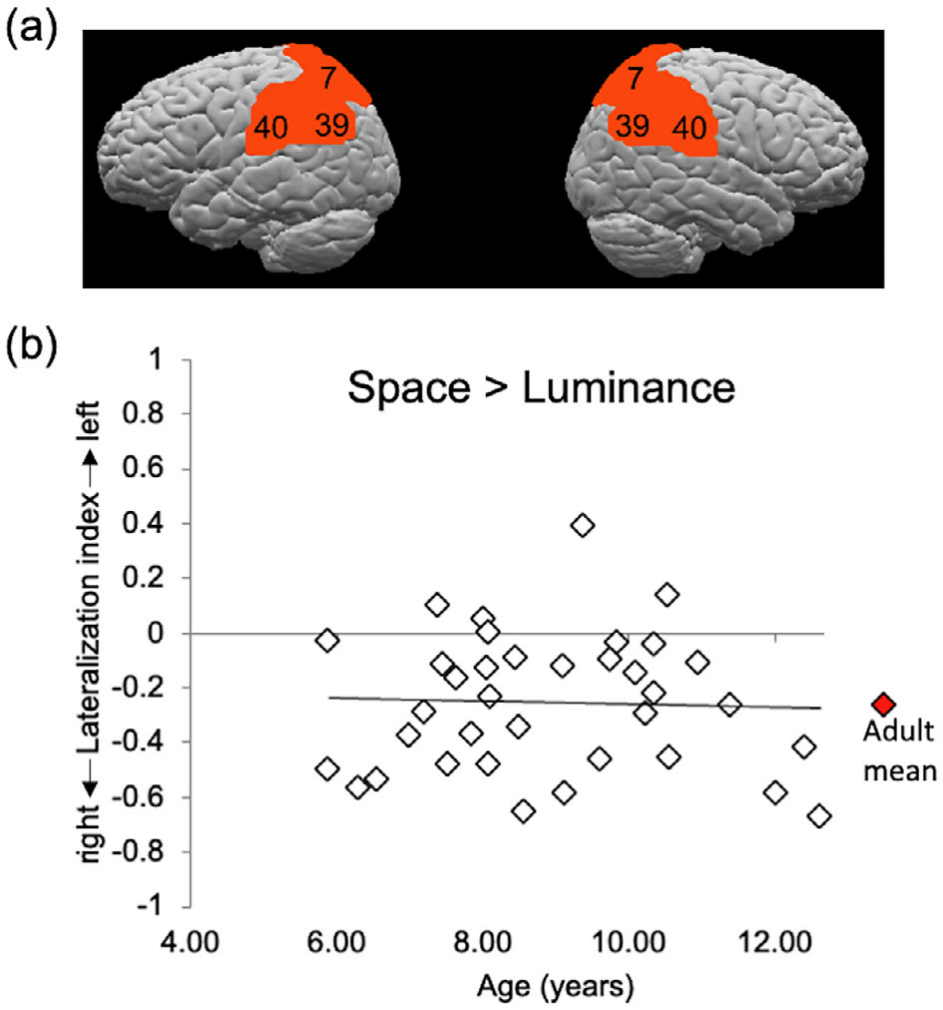
Lateralization analysis. (a) The anatomically‐defined bilateral posterior parietal ROI encompassing Brodmann areas 7, 40, and 39 was used to analyze lateralization of function in children for the line bisection task. (b) Lateralization index values for individual child participants for the contrast Spatial > Luminance. The linear trendline of the data is shown. For illustration purposes, the adult mean LI for this same task (Seydell‐Greenwald et al. 2019) is shown in red. This illustrates that the LIs of children between ages 5 and 12 tend to be right‐lateralized and are distributed around the adult mean, with no apparent lateralization increase over age.

To test for a developmental trend, LIs were computed for individual participants (see Methods). We examined LI within the anatomically‐defined bilateral posterior parietal ROI that comprised BAs 7, 40, and 39 (see Figure 5a). Figure 5b shows the LIs for individual children over age for the contrast of Spatial > Luminance. Overall, group activation was right‐lateralized, with a mean LI of −0.25 (SE = 0.04). Mean and range of LIs are in excellent agreement with those observed in adults performing this same task (Seydell‐Greenwald et al. 2019). Pearson product‐moment correlation coefficients (two‐tailed) were computed to examine the relationships between LI and age. There was no significant negative correlation between LI and age, *r* = –0.038.

## 4 Discussion

In the present study we asked whether certain dominant patterns of hemispheric lateralization shown among adults are prominent from early development onward, or whether lateralization patterns change significantly over time. The answer to this question is important for our understanding of how lateralization patterns come to be the way they are in adulthood and whether there are general principles of lateralization development that span different types of cognitive functions. Our work was inspired by recent studies on the development of hemispheric lateralization for language, which shows that although language (specifically, syntax and sentential semantics) tends overwhelmingly to be left‐lateralized in healthy adults, its lateralization profile undergoes significant developmental change between the ages of 4 and 13 years, starting out more bilateral and becoming strongly left‐lateralized by late childhood/early adolescence (Berl et al. 2014; Olulade et al. 2020). These findings raise the question of whether this same developmental pattern is also observed in the realm of spatial functions, which classically have been assumed to be right‐lateralized. We tested this question using fMRI to examine brain activation in children between the ages of 5 and 12 years, using the line bisection/ Landmark task, which is known to robustly and reliably activate the right inferior and superior parietal lobes in healthy adults (Seydell‐Greenwald et al. 2019; see Badzakova‐Trajkov et al. 2016, for a review).

In the Spatial condition of our line bisection task, children judged whether the top or bottom of a segmented line was longer. In the control Luminance condition, they judged whether the top or bottom of the line was brighter. Because we aimed to investigate changes in lateralization over a broad developmental period and wanted to avoid the potential influence of task difficulty on activation patterns (Just et al. 1996; Murphy and Garavan 2004; Yeatman et al. 2010), the “Line Game” was designed to be relatively easy to carry out for all ages. Participants showed high accuracy in behavioral performance.

The crucial question, however, concerned the lateralization profile for this task over age and specifically whether the adult pattern of right lateralization emerges at the earliest ages tested, or whether there is a developmental change from initially more bilateral to ultimately adult‐level lateralized, as has been shown for language (Olulade et al. 2020). The results showed the former: Although there was considerable variability in LIs across participants—as was also true of adults in a prior study (Seydell‐Greenwald et al. 2019)—right‐lateralized LIs were already observed in the youngest participants tested here, and there were no significant effects of age on strength and lateralization of activation during the Spatial task condition. That is, within the age range tested here (5−12 years), we found stable lateralization to the right hemisphere for the line bisection task.

Our findings show a clear contrast with the development of lateralization for language, as found by Berl et al. (2014) and Olulade et al. (2020). In both studies, the youngest children (5−7 years old) showed bilateral activation in a language comprehension task requiring that they indicate whether sentences defining a familiar noun were true. Language activation occurred in the typical regions of the left hemisphere known to support language in adults as well as in the homotopic regions of the right hemisphere. This bilateral activation pattern can help explain the finding that children who have sustained perinatal strokes to the left hemisphere can develop language, with high levels of performance in adolescence on a range of tasks (Newport et al. 2017), and no differences between those who have sustained left versus right strokes earlier in life (Stiles et al. 2012). The most telling result is that children who have sustained perinatal damage to the left hemisphere show fully developed language as adolescents and young adults, indicating that both right and left hemispheres are initially capable of supporting language function.

Our findings also contrast with the observation of developmental changes in the age range tested here for face processing. A number of studies have found that face processing is bilateral in infancy (Kosakowski et al. 2022) and during early childhood (Behrmann and Plaut 2020; Dundas et al. 2014; Lochy et al. 2019), and then becomes robustly right‐lateralized over development, in conjunction with the onset of reading and the formation of the Visual Word Form Area (VWFA). Additional studies have shown that, indeed, the process of learning to read contributes to the establishment of the VWFA, its response to letters and words, and in turn, decreases neural response to faces in these and neighboring areas (Cantlon et al. 2011; Dehaene et al. 2015). The results of this process are that letter and word recognition are established as a left‐lateralized function, while perception of faces becomes right‐lateralized. This mechanism for establishing lateralization (for faces) is thus entwined with specific experience, that is, learning to read.

We also note that increases in right‐lateralization among children between 7 and 20 years of age have been found for other visual‐spatial tasks (Everts et al. 2009; Lidzba et al. 2013; Lidzba et al. 2006). In those studies, the tasks required visual search, with the test condition requiring participants to search for a missing detail in a complex figure and the control condition requiring them to judge whether two complex figures had the same orientation. Both visual search and line bisection are commonly used as measures of visual attention, which has long been conceived of as a function for which the right hemisphere is dominant, based on the observation that contralesional attentional neglect is more common after right than left hemisphere lesions in adults (Heilman and van den Abell 1980). However, it is important to note that more recent theories of attention suggest that visual attention networks, especially in the parietal regions activated in our task, are much more bilateral than previously thought (Shulman et al. 2010), with weighting between the hemispheric contributions that are responsive to task and individual differences and directly linked to the slight spatial biases observed in line bisection performance of neurologically healthy individuals (Szczepanski and Kastner 2013).

Given that other visual‐spatial tasks appear to undergo increases in right‐lateralization within the age range that we have examined, why did we not observe the same developmental trend? Potential explanations for this fall into two categories: (1) There may be a change in lateralization, but it occurs at younger ages than we tested, and (2) there truly is no change in lateralization for this particular task; that is, line bisection is right‐lateralized from infancy onward.

Evidence regarding either of these possibilities is quite limited, because children younger than 5 years of age have not been tested in tasks similar to ours. For this reason, we regard it as an open empirical question. However, in a recent study, Nava et al. (2022) examined gaze patterns in 4−5‐month‐olds, who were trained to fixate the center of a horizontal line (by providing a vertical blinking line at its midpoint) and then tested without the blinking bar. Results showed that the infants fixated to the left of the true midpoint, consistent with well‐known patterns of “pseudoneglect” shown by healthy adults, which is hypothesized to reflect right‐hemisphere dominance for attention processes. This might suggest that the line bisection task is indeed right‐lateralized from infancy on. However, the pattern of leftward looking was found only for horizontal but not vertical lines (which elicited a pattern of rightward looking, perhaps consistent with left hemisphere involvement). At present, these infant studies provide suggestive support for the idea that the line bisection task engages both hemispheres, with specific contributions from each perhaps differing depending on the specific stimuli and methods used.

There is also a larger literature suggesting that both hemispheres are somewhat involved in the line bisection task. First, the activations observed among adults for our task are only weakly right‐lateralized even at the group level, and much weaker and less consistently lateralized at the individual level (Seydell‐Greenwald et al. 2019). This compares with the highly consistent and strongly left‐lateralized individual activations that we usually observe for sentence comprehension in adults (e.g., Seydell‐Greenwald et al. 2020). The relatively weak lateralization in both the children we tested and adults is consistent with the idea of bilateral influences and large interindividual variability in lateralization for the line bisection task (Szczepanski and Kastner 2013). Given such weak lateralization and large variability of lateralization in adults, it is perhaps not surprising that a developmental trend could not be detected in the present study, even if such a trend exists. In this context it is also worth noting that we have previously found bilateral activation of the same parietal regions for a complex visual‐spatial construction task requiring judgments of whether two figures can be combined to form a third, in both adults (Seydell‐Greenwald et al. 2017) and children (Ferrara et al. 2020), with no change over ages 5–11. As we have argued (Ferrara et al. 2020), visual‐spatial tasks as a whole vary considerably and can require quite different computations and therefore might be expected to elicit different patterns of lateralization over development.

Before closing, we wish to point out that the question of when different visual‐spatial functions come to show mature lateralization patterns is important not only for our understanding of whether “space” as a whole is right‐lateralized from infancy or develops over time, but also for our understanding of how brain injury early in development might affect ultimate developmental outcomes in childhood and adolescence. As we have noted, in the case of language, young children show more bilateral patterns of activation to language, reaching adult levels of left‐lateralization by adolescence. These findings help to explain the robust preservation of language in individuals who have sustained perinatal strokes: that is, if both left and right hemispheres are initially involved in language, then following a left hemisphere stroke in infancy, the healthy right hemisphere should be capable of supporting language, as shown by Newport et al. (2022). Understanding the developmental profile for lateralization of visual‐spatial tasks like the line bisection task can similarly help us understand outcomes for visual‐spatial functions following perinatal stroke to the right hemisphere, a topic which we are pursuing.

In conclusion, the present study uses an adapted version of the “Landmark Task” to investigate the lateralization profile in children. We find that children between 5−12 years of age show right‐lateralized parietal activation, similar to that shown by adults, with no effects of age within the range we tested. Our findings raise new questions about this pattern of lateralization and its development and suggest new avenues of research to examine developmental trajectories of a wide range of functions, as we seek to discover whether lateralization over development shares common principles for different tasks and domains. As we noted earlier, it is important to recognize that not all spatial tasks show the same profiles over development (e.g., visual search, Everts et al. 2009, vs. visual‐spatial construction, Ferrara et al. 2020). In future work, it will thus be critical to consider the computational requirements of different spatial tasks and the ways in which these may engage the potential representational biases of the two hemispheres.

## Conflicts of Interest

The authors declare no conflicts of interest.

## Supporting information




**Supporting File 1**: desc70060‐sup‐0001‐SuppMat.docx

## Data Availability

The data that support the findings of this study are available from the corresponding author upon reasonable request. The data are not publicly available due to privacy or ethical restrictions.

## Endnotes

1 As a check to ensure that different levels of smoothing did not impact our results, data were also analyzed using an 8 mm FWHM Gaussian kernel (which is the level of spatial smoothing used to analyze adult data in Seydell‐Greenwald et al. 2019). Findings were comparable and not qualitatively different for 6 and 8 mm smoothing.

2 In addition to this activation, Seydell‐Greenwald et al. (2019) previously found that adults also showed activation in the superior parietal lobe (SPL, BA 7) for the contrast of Spatial > Luminance. As a group, children showed activation in the same area; however, this was only evident at a more lenient threshold (*p* < 0.003). This may suggest that reliance on these areas is common in adults, but not as consistent in children.
