# Supplementary material for: Developmental Changes in Neural Lateralization for Visual‐Spatial Function? Evidence From a Line‐Bisection Task
Source: Dev Sci. 2025 Aug 9;28(5):e70060. doi: 10.1111/desc.70060 (PMC12335016; doi:10.1111/desc.70060)
Supplement: Supplementary file 1 — Supporting File 1: desc70060‐sup‐0001‐SuppMat.docx [file DESC-28-e70060-s001.docx]

**Supplementary Information**

*Stimuli: Functional MRI Paradigm*

Figure S1 below illustrates the instruction screens that were shown at the start of each condition block in the scanner.

Figure S1. Instruction screens that preceded each block.

A) Instruction screen for Spatial blocks. Participants heard the question, “Is the line longer on the top or the bottom?”

B) Instruction screen for Luminance blocks. Participants heard the question, “Is the line brighter on the top or the bottom?”

*Regions of Interest*

*Anatomical ROI*

Figure S2 illustrates the anatomically-defined ROI. This is the same ROI that was used for analyses in a previous fMRI study of visual line bisection in healthy adults (Seydell-Greenwald et al., 2019). The regions included within this ROI are commonly cited in the literature as important for visual-spatial processing (e.g., Bogen & Gazzaniga, 1965; Zacks, 2008; Tomasino & Gremese, 2016; Brain, 1941; McFie et al., 1950; Hecaen et al., 1956; Vallar, 1998; Fierro et al., 2000; Fink et al., 2000; Çiçek et al., 2009; Cavézian et al., 2012), and constitute the same ROI as used in analyzing lateralization of function among healthy adults who carried out the same line bisection task described in the present paper (Seydell-Greenwald et al., 2019). Analysis of the child data using this ROI allows us to determine whether the current study reveals changes in lateralization over age and to compare any such changes to patterns exhibited by adults in the previous study.


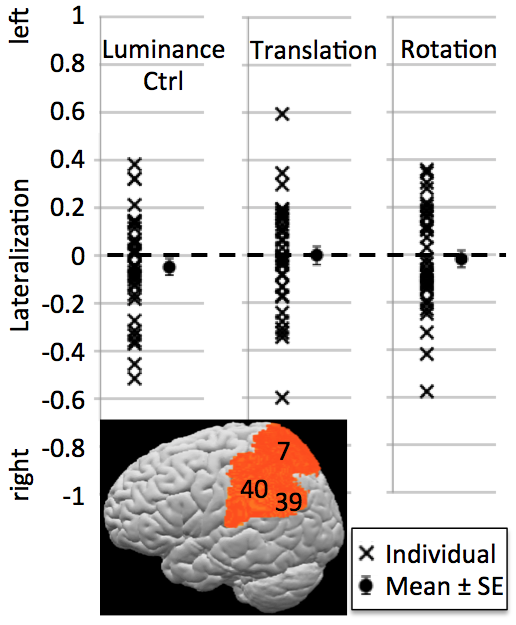


Figure S2. Lateralization Indices (LI) were computed within a bilateral posterior parietal ROI comprising BAs 7 (SPL), 40 (IPL), and 39 (near the junction of the temporal, occipital, and parietal lobe).

*Functional ROI*

In addition to the anatomically-defined ROI described above, we also created an ROI based upon adult functional activation in the same task (Seydell-Greenwald et al., 2019), with the aim of investigating activation within a smaller and more specific parietal area. Whole-brain analyses revealed highly similar areas of activation between children and adults in the right inferior parietal lobe (IPL). Figure S3 below illustrates the overlap of child data (the present study, shown in red) and adult data (Seydell-Greenwald et al., 2019, shown in blue) for the contrast of Spatial > Luminance. The area of overlap of the child and adult activation is shown in green. This high degree of overlap allowed us to create an independent ROI within which to evaluate child activation while avoiding the circularity of double-dipping (Kriegeskorte et al., 2009). To define the ROI, adult data were first re-analyzed to match the preprocessing methods of the present study. They were then aligned in Talairach space for comparison to the child data. An adult activation map (p < .001, cluster-size threshold of k < 0.05) was created for the contrast of Spatial > Luminance and an ROI was defined based upon a significant cluster of activation in the right IPL). To create a bilateral version of this ROI, activation in the adult right IPL was flipped over the midline to create a homotopic cluster in the left IPL, and both right and left ROIs were combined. Table S1 below provides further details about the functional ROI that was created based upon the adult data.


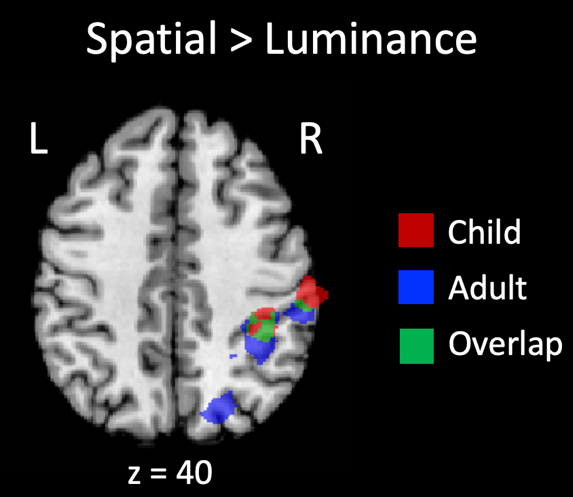


Figure S3. Overlap of child (present study) and adult (Seydell-Greenwald et al., 2019) whole-brain activation for the contrast of Spatial > Luminance. Child data are shown in red, adult data are shown in blue, and the area of overlap of the child and adult activation is shown in green. Child activation is nearly entirely subsumed by the adult activation. Adult and child data were analyzed according to the same preprocessing steps (see Methods). Activation maps are overlaid on the Colin27 brain template transformed into Talairach space and thresholded at *p* < .001 single-voxel threshold combined with a k < 0.05 cluster-size threshold.

Table S1. Details for the adult-defined functional ROI, created from data from adult activation in the same study (Seydell-Greenwald et al., 2019). This ROI was based upon significant adult group activation for the contrast of Spatial > Luminance in the inferior parietal lobe. The Peak Talairach coordinates of the adult activation, Peak t, and extent of the activation cluster (mm^3^) are provided below. IPL; inferior parietal lobe, BA; Brodmann area.

| Location description | BA | Peak Tal coords | Center of Gravity | Peak t-value | Average t-value | Average p-value | Cluster extent (mm^3^) |
| --- | --- | --- | --- | --- | --- | --- | --- |
| IPL | 40 | 53, -29, 42 | 45, -33, 43 | 7.13 | 3.77 | 0.015000 | 10574 |

*Analyses of Activation within ROIs*

To investigate the response activation profile within an ROI, the average percent signal change across all voxels in the ROI was extracted separately for each child participant and condition. This was done separately for the left and the right hemispheres to evaluate potential differences in activation. Multiple linear regressions were calculated to predict activation (percent signal change) based on age, accuracy, and RT for behavioral performance in the scanner. Separate linear regressions were calculated for the left hemisphere and for the right, in recognition of the possibility that activation may be significantly related to age or performance in one hemisphere but not the other.

For the adult-defined functional ROI, a significant regression equation was found for the Spatial condition in the left hemisphere, *F*(3, 32) = 6.30, *p* = .002, with an *R^2^* of .37. Both accuracy (*p* = .031) and RT (*p* = .038), but not age, emerged as significant predictors of left parietal activation in the spatial condition, see Table S2. Nonsignificant regression equations were found for the Spatial condition in the right hemisphere (*F*(3, 32) = 0.62, *p* = .61, *R^2^* of .06) as well as for the Luminance condition in both the left (*F*(3, 32) = 0.33, *p* = .80, *R^2^* of .03) and the right (*F*(3, 32) = 0.10, *p* = .96, *R^2^* of .01) hemispheres. The absence of significant age effects is consistent with that found for the anatomically-defined parietal ROI, as reported in the main text.

Table S2. Summary of the linear regression equation for variables predicting activation (percent signal change) for the Spatial condition in the adult functionally-defined ROI in the left hemisphere. Age was not a significant predictor of activation.

| Variable | *B* | *SE B* | *β* | *p* |
| --- | --- | --- | --- | --- |
| Age | 0.02 | 0.02 | 0.17 | 0.334 |
| Accuracy | <0.001 | <0.001 | -.40 | 0.031* |
| RT | 1.13 | 0.52 | 0.31 | 0.038* |
| * *p* < .05. |  |  |  |  |

*Whole-brain analyses: Individual Participant Maps*

Inspection of the individual participant maps revealed that some participants showed activation in right hemisphere parietal areas as well as in the homotopic areas of the left hemisphere, whereas others showed significant activation only in the right hemisphere. Figure S4 below illustrates individual child participant maps ranging in age from 5 years, 10 months to 10 years, 2 months. Overall, there is no systematic relationship between age and whether a participant displays a bilateral or more right-lateralized (adult-like) patterns of activation.


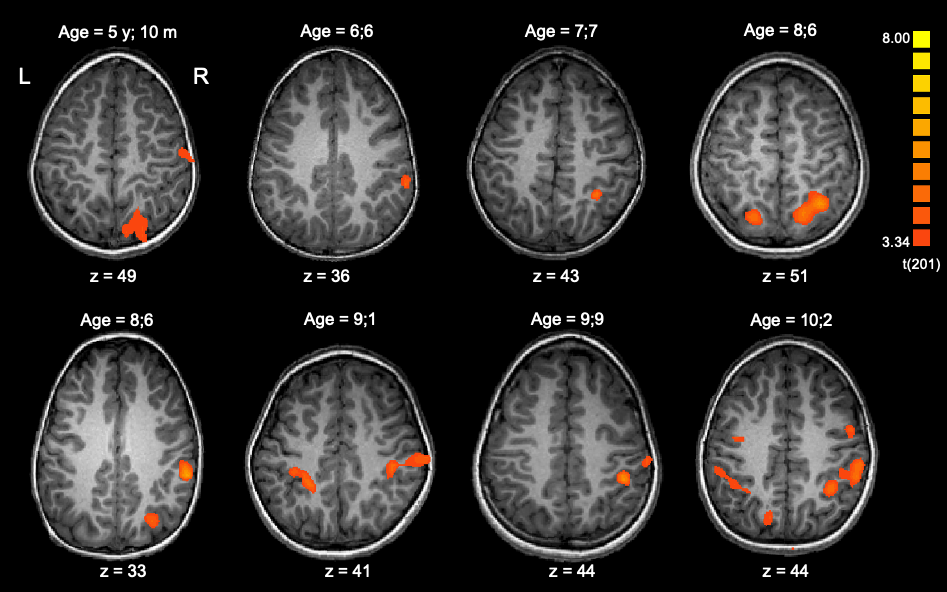


Figure S4. Areas displaying significantly stronger activations for the contrast of Spatial > Luminance in a sample of individual child participants. Participants are ordered by age, youngest to oldest (age; years). Activation maps are overlaid on the individual participant’s MPRAGE, transformed into Talairach space, and thresholded at *p* < .001 single-voxel threshold combined with a k < 0.05 cluster-size threshold.

*Laterality indices*

Laterality indices (LIs) were computed for individual participants. In addition to the LI analyses based on the anatomical ROI (reported in the main text), we also carried out a second analysis using a functional ROI that was based on adult group-level activation shown in the dataset collected by Seydell-Greenwald et al. (2019). Analyses of LI within this adult-defined functional ROI replicated the children’s results for the larger anatomical parietal ROI (Figure S5). Participants had a mean LI of −.26 (SE = .04). There was no significant correlation between LI and age (*r* = −.28, *p* = .010).


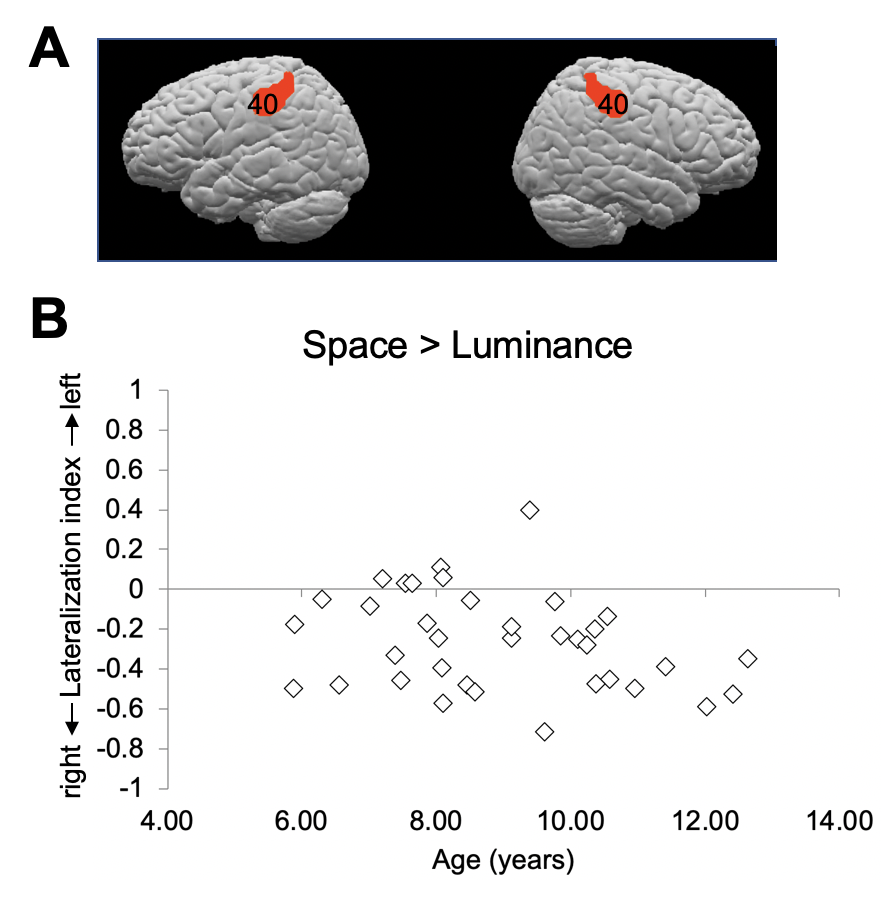


Figure S5. Laterality analysis using a functional ROI defined on the basis of healthy adult data from Seydell-Greenwald et al. (2019).

A) A bilateral inferior parietal ROI was defined using adult activation for the contrast of Spatial > Luminance.

B) As was found for the anatomically-defined ROI, laterality indices calculated within this more constrained functional ROI showed no systematic changes across the age range tested.

*References*

Bogen, J. E., & Gazzaniga, M. S. (1965). Cerebral commissurotomy in man: Minor hemisphere dominance for certain visuospatial functions. *Journal of Neurosurgery, 23,* 394–399. [https://doi.org/10.3171/jns.1965.23.4.](https://doi.org/10.3171/jns.1965.23.4.0394)[0394](https://doi.org/10.3171/jns.1965.23.4.0394)

Brain, W. R. (1941). Visual orientation with special reference to lesions of the right cerebral hemisphere. Brain: A Journal of Neurology, 64, 244–272. [https://doi.org/10.1093/brain/64.4.244](https://psycnet.apa.org/doi/10.1093/brain/64.4.244)

Cavézian, C., Valadao, D., Hurwitz, M., Saoud, M., & Danckert, J. (2012). Finding center: Ocular and fMRI investigations of bisection and landmark task performance. *Brain Research, 1437,* 89–103. <https://doi.org/10.1016/j.brainres.2011.12.002>

Çiçek, M., Deouell, L. Y., & Knight, R. T. (2009). Brain activity during landmark and line bisection. *Frontiers in Human Neuroscience, 3,* 7. <https://doi.org/10.3389/neuro.09.007.2009>

Fierro, B., Brighina, F., Oliveri, M., Piazza, A., La Bua, V., Buffa, D., & Bisiach, E. (2000). Contralateral neglect induced by right posterior parietal rTMS in healthy subjects. *Neuroreport, 11,* 1519–1521. [https://doi.org/10.1097/](https://doi.org/10.1097/00001756-200005150-00031)[00001756-200005150-00031](https://doi.org/10.1097/00001756-200005150-00031)

Fink, G. R., Marshall, J. C., Shah, N. J., Weiss, P. H., Halligan, P. W., Grosse-Ruyken, M., Ziemons, K., Zilles, K., & Freund, H. J. (2000). Line bisection judgments implicate right parietal cortex and cerebellum as assessed by fMRI. *Neurology, 54,* 1324–1331. [https://doi.org/10.1212/WNL.54.6.](https://doi.org/10.1212/WNL.54.6.1324)[1324](https://doi.org/10.1212/WNL.54.6.1324)

Hecaen, H., Penfield, W., Bertrand, C., & Malmo, R. (1956). The syndrome of apractognosia due to lesions of the minor cerebral hemisphere. *AMA Archives of Neurology & Psychiatry, 75*(4), 400–434. [https://doi.org/10.](https://doi.org/10.1001/archneurpsyc.1956.02330220064007)[1001/archneurpsyc.1956.02330220064007](https://doi.org/10.1001/archneurpsyc.1956.02330220064007)

Kriegeskorte, N., Simmons, W. K., Bellgowan, P. S. F., & Baker, C. I. (2009). Circular analysis in systems neuroscience: the dangers of double dipping. *Nature Neuroscience, 12,* 535–540. <https://doi.org/10.1038/nn.2303>

McFie, J., Piercy, M. F., & Zangwill, O. L. (1950). Visual-spatial agnosia associated with lesions of the right cerebral hemisphere. *Brain, 73,* 167–190. <https://doi.org/10.1093/brain/73.2.167>

Seydell-Greenwald, A., Pu, S. F., Ferrara, K., Chambers, C. E., Newport, E. L., & Landau, B. (2019). Revisiting the landmark task as a tool for studying hemispheric specialization: What’s really right? *Neuropsychologia, 127,* 57–65. <https://doi.org/10.1016/j.neuropsychologia.2019.01.022>

Tomasino, B., & Gremese, M. (2016). Effects of stimulus type and strategy on mental rotation network: An activation likelihood estimation metaanalysis. Frontiers in Human *Neuroscience, 9,* Article 693. <https://doi.org/10.3389/fnhum.2015.00693>

Vallar, G., & Perani, D. (1986). The anatomy of unilateral neglect after right hemisphere stroke lesions. A clinical/CT-scan correlation study in man. *Neuropsychologia, 24,* 609–622. [https://doi.org/10.1016/0028-3932(86)](https://doi.org/10.1016/0028-3932(86)90001-1)[90001-1](https://doi.org/10.1016/0028-3932(86)90001-1)

Zacks, J. M. (2008). Neuroimaging studies of mental rotation: A metaanalysis and review. *Journal of Cognitive Neuroscience, 20,* 1–19. <https://doi.org/10.1162/jocn.2008.20013>
